# The importance of the linkage between national and international research funders to support malaria elimination

**DOI:** 10.1186/1475-2875-11-S1-O4

**Published:** 2012-10-15

**Authors:** Hassan Mshinda

**Affiliations:** 1 Tanzania Commission for Science and Technology, Tanzania

## 

Abstract not submitted

